# Supplementary material for: Polymer Networks Synthesized from Poly(Sorbitol Adipate) and Functionalized Poly(Ethylene Glycol)
Source: Gels. 2021 Feb 20;7(1):22. doi: 10.3390/gels7010022 (PMC8006044; doi:10.3390/gels7010022)
Supplement: Supplementary file 1 [file gels-07-00022-s001.pdf]

Supplementary Materials:

Polymer Networks Synthesized from Poly(sorbitol adipate) and Functionalized Poly(ethylene glycol)

Haroon Rashid[1,2], Yury Golitsyn[3], Muhammad Humayun Bilal[1], Karsten Mäder[2], Detlef Reichert[3], Jörg Kressler[1,*]

1 Department of Chemistry, Martin Luther University Halle-Wittenberg, D-06120 Halle (Saale), Germany; haroon.rashid@student.uni-halle.de (H.R.); muhammad.bilal@chemie.uni-halle.de (M.H.B.)

2 Institute of Pharmacy, Martin Luther University Halle-Wittenberg, D-06120 Halle (Saale), Germany; karsten.maeder@pharmazie.uni-halle.de (K.M.)

3 Department of Physics, Martin Luther University Halle-Wittenberg, D-06120 Halle (Saale), Germany;

yury.golitsyn@physik.uni-halle.de (Y.G.); detlef.reichert@physik.uni-halle.de (D.R.)

* Correspondence: joerg.kressler@chemie.uni-halle.de (J.K.); Tel.: +49-345-552-5800

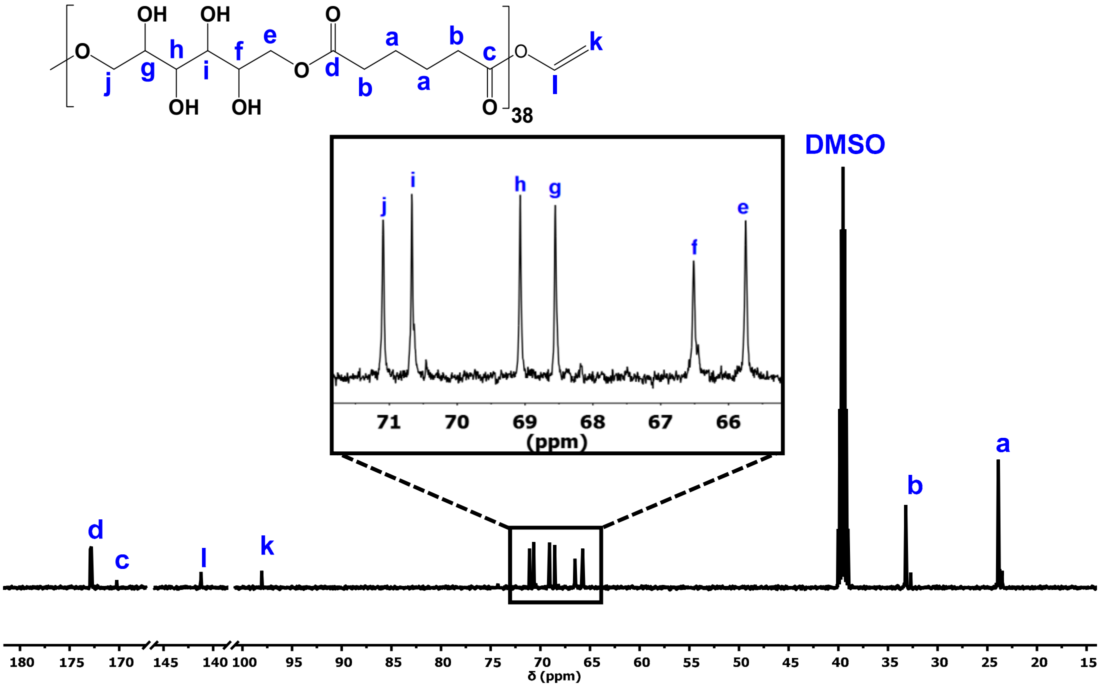

Figure S1. ^{13}C NMR spectrum of poly(sorbitol adipate) measured at 27°C using DMSO-d_6 as solvent.

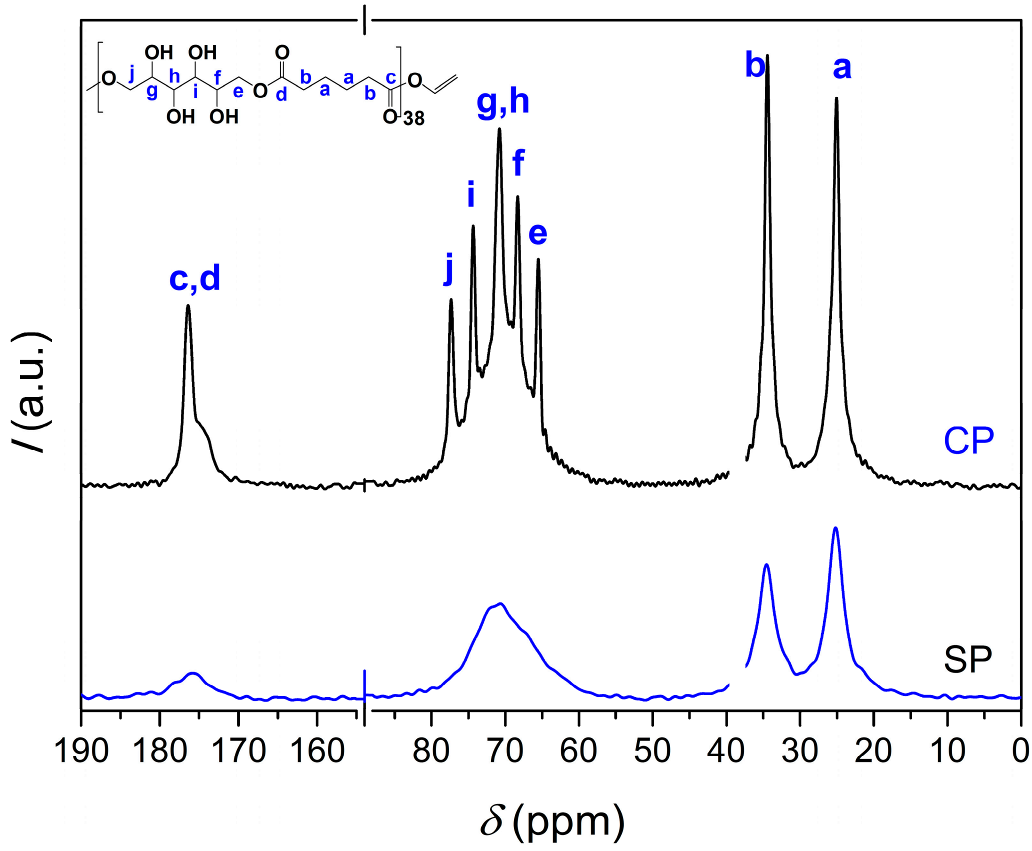

Figure S2. ^{13}C CP (top) and SP (bottom) MAS spectra of poly(sorbitol adipate). PSA is a highly viscous substance with low mobility. The SP experiment, therefore, does not provide spectral resolution. The CP experiment shows a well resolved spectrum.

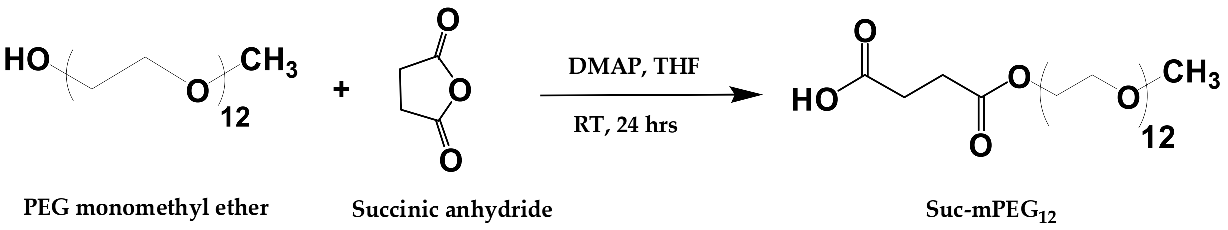

Scheme S1. Synthesis scheme of mPEG12-Suc

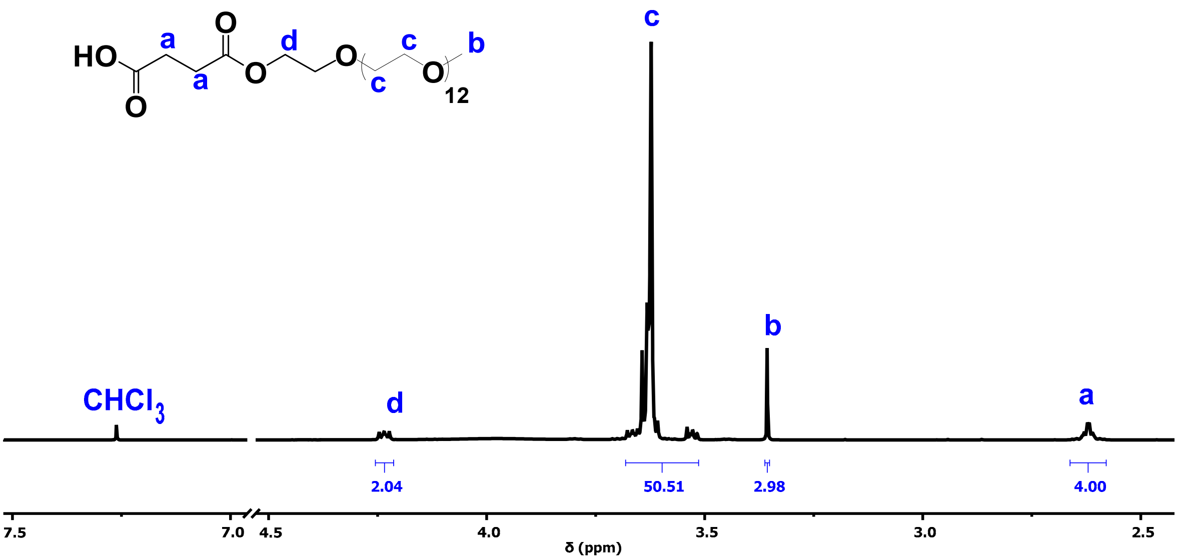

Figure S3. ^{1}H NMR spectrum of mPEG12-Suc measured at 27°C using $CDCl_3$ as solvent.

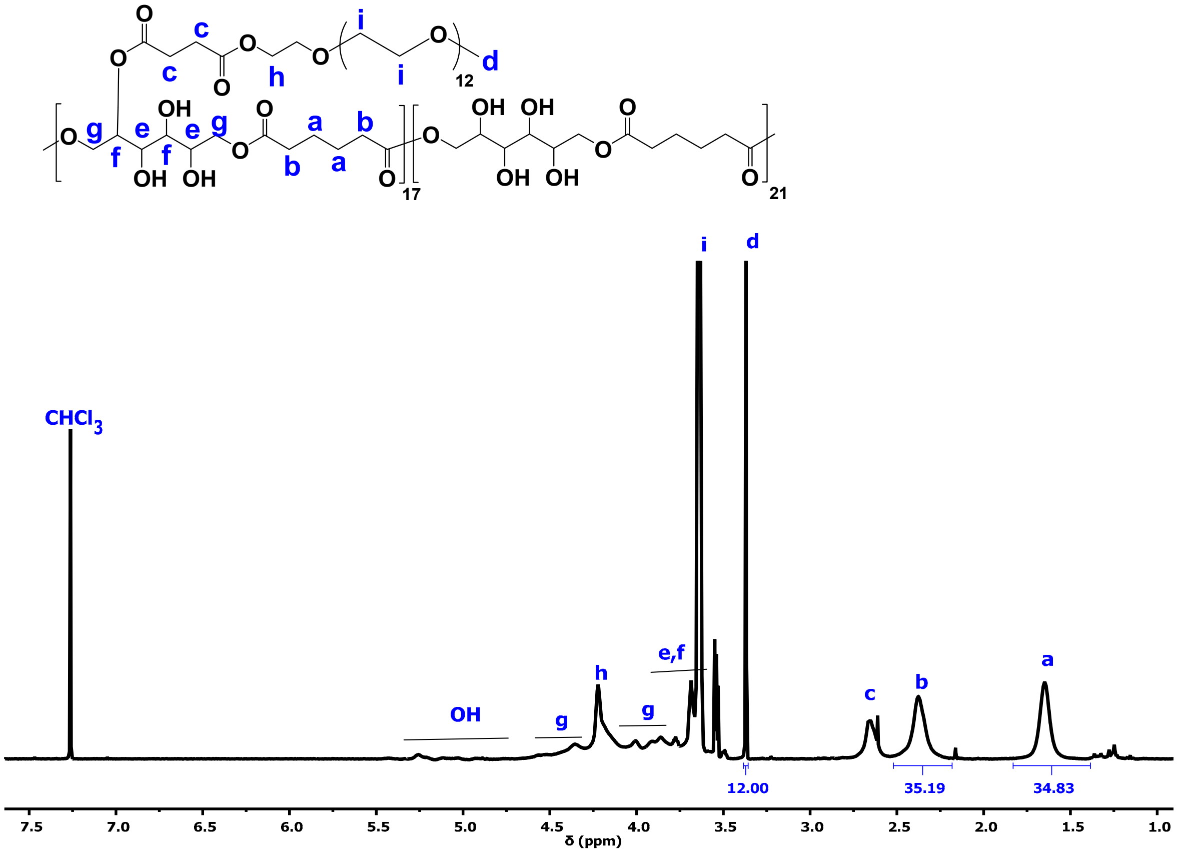

Figure S4. ¹H NMR spectrum of PSA-*g*-mPEG$_{12}$ measured at 27°C using $CDCl_3$ as solvent.

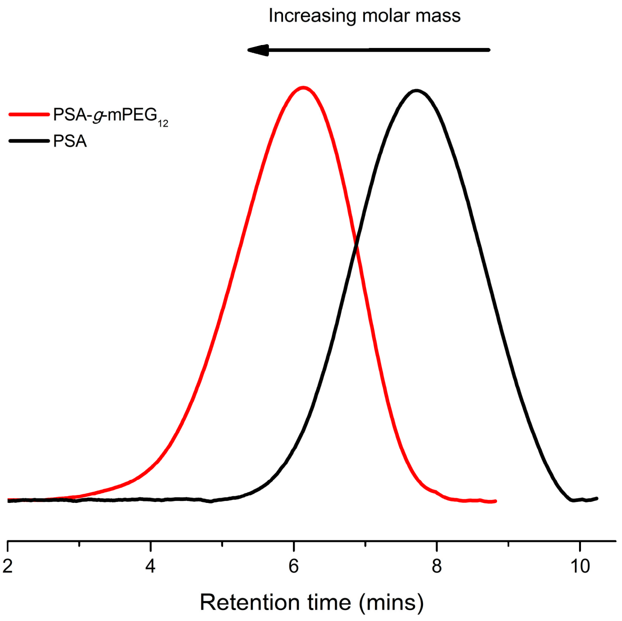

Figure S5. GPC traces of PSA before and after modification with mPEG$_{12}$-Suc.

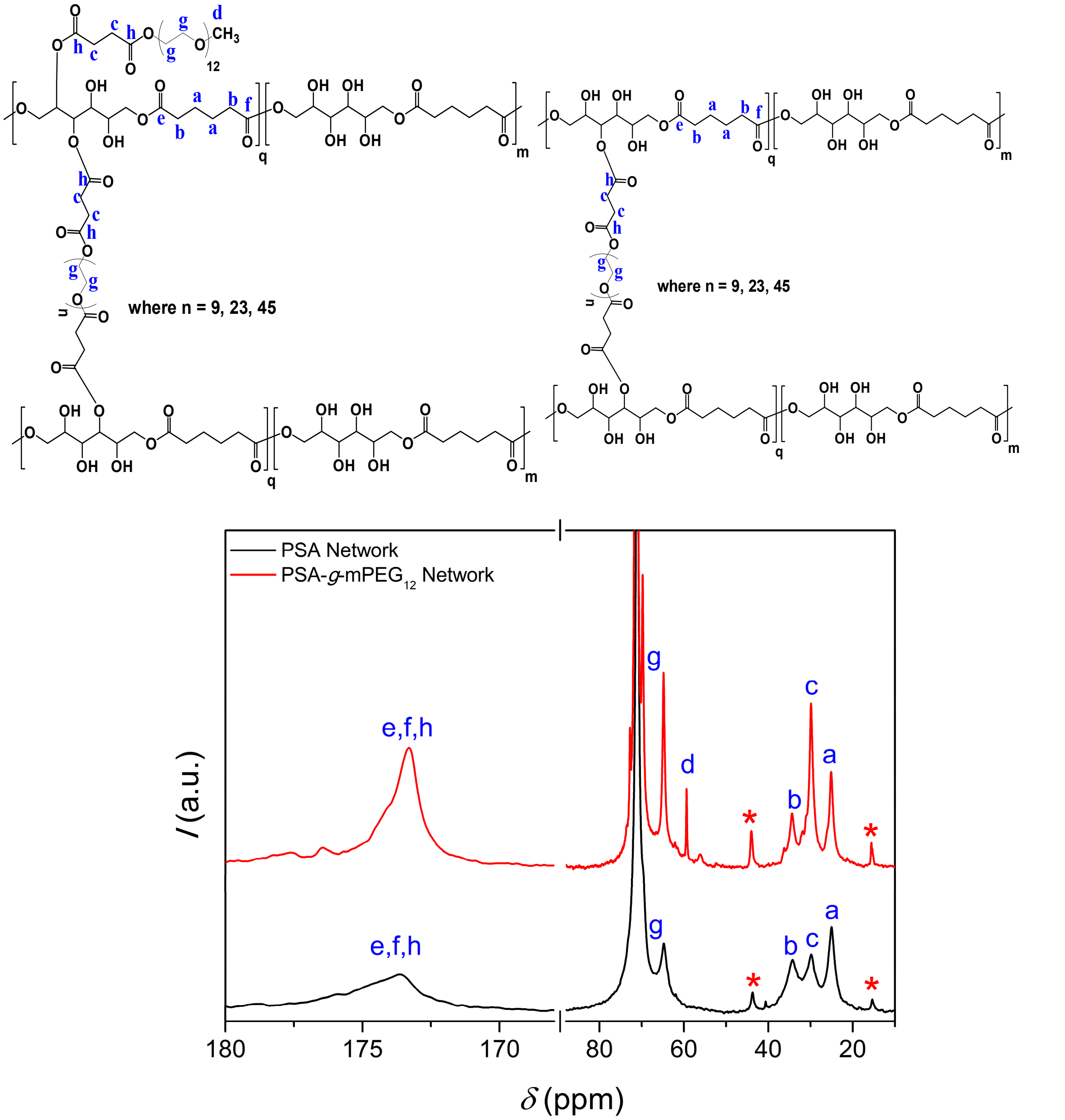

Figure S6. ^{13}C SP spectra of PSA and PSA-*g*-mPEG$_{12}$ networks with a Suc-PEG$_9$-Suc cross-linker. Both spectra show incorporation of identical cross-linker's succinyl peaks at 30 ppm while carbon peaks from ethylene glycol part are appearing around 70 ppm. Difference between both type of networks is that PSA-*g*-mPEG$_{12}$ network shows a carbon peak of methyl at 58 ppm from mPEG$_{12}$ which is absent in the PSA network. Furthermore, from PSA-*g*-mPEG$_{12}$ network spectra, carbon peaks at around 30 ppm and 173 ppm shows greater intensity due to the grafted chains of mPEG$_{12}$-Suc. There are two unknown extra carbon peaks appearing around 15 ppm and 45 ppm, marked by an asterisk. One possible reason can be peak splitting of PEG based cross-linker after formation of the network.

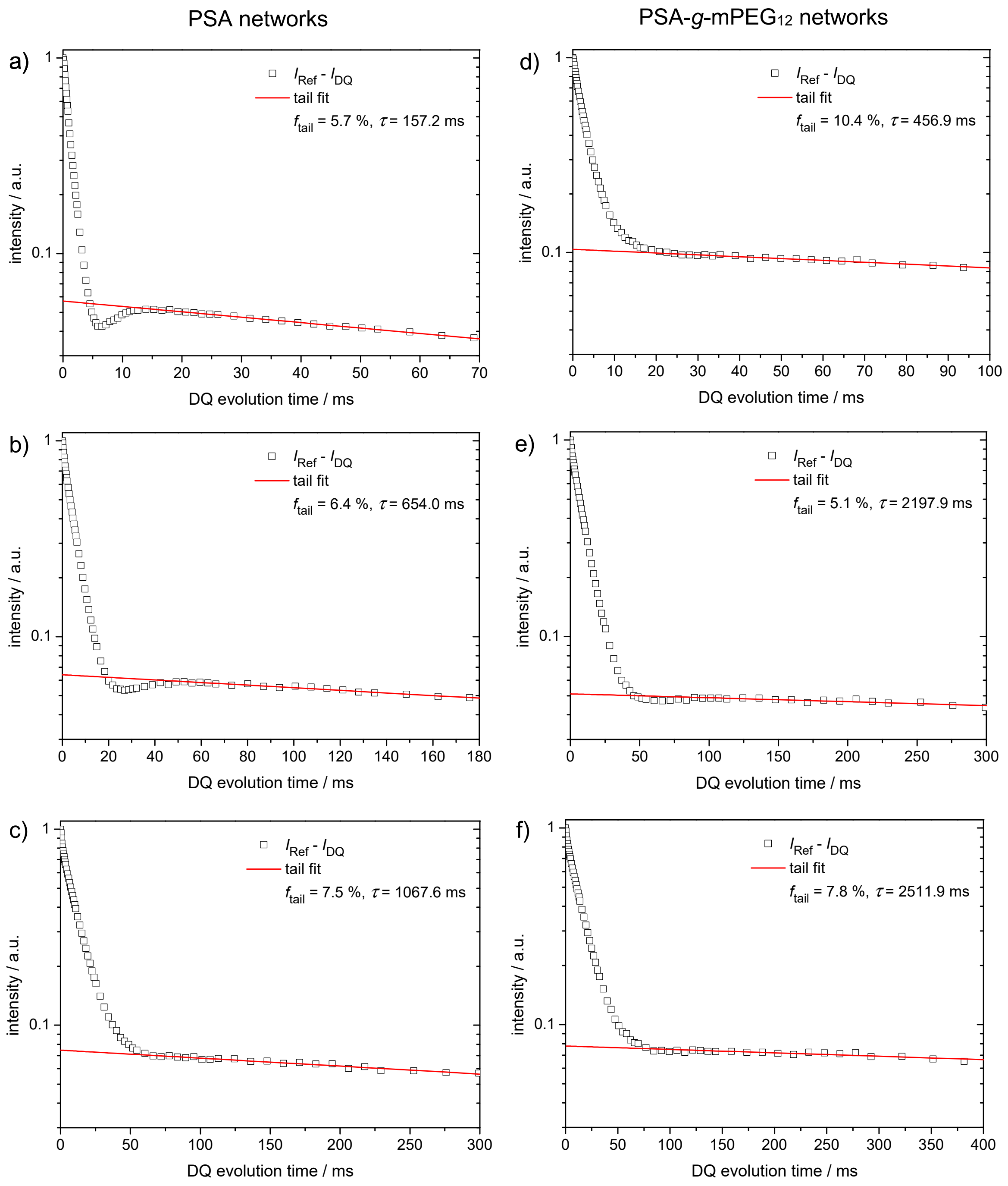

Figure S7. Determination of the tail fraction through ^{1}H DQ NMR for (a-c) PSA networks cross-linked with the Suc-PEG$_n$-Suc (where n = 9, 23, 45) and (d-f) PSA-*g*-mPEG$_{12}$ networks cross-linked with the Suc-PEG$_n$-Suc (where n = 9, 23, 45).

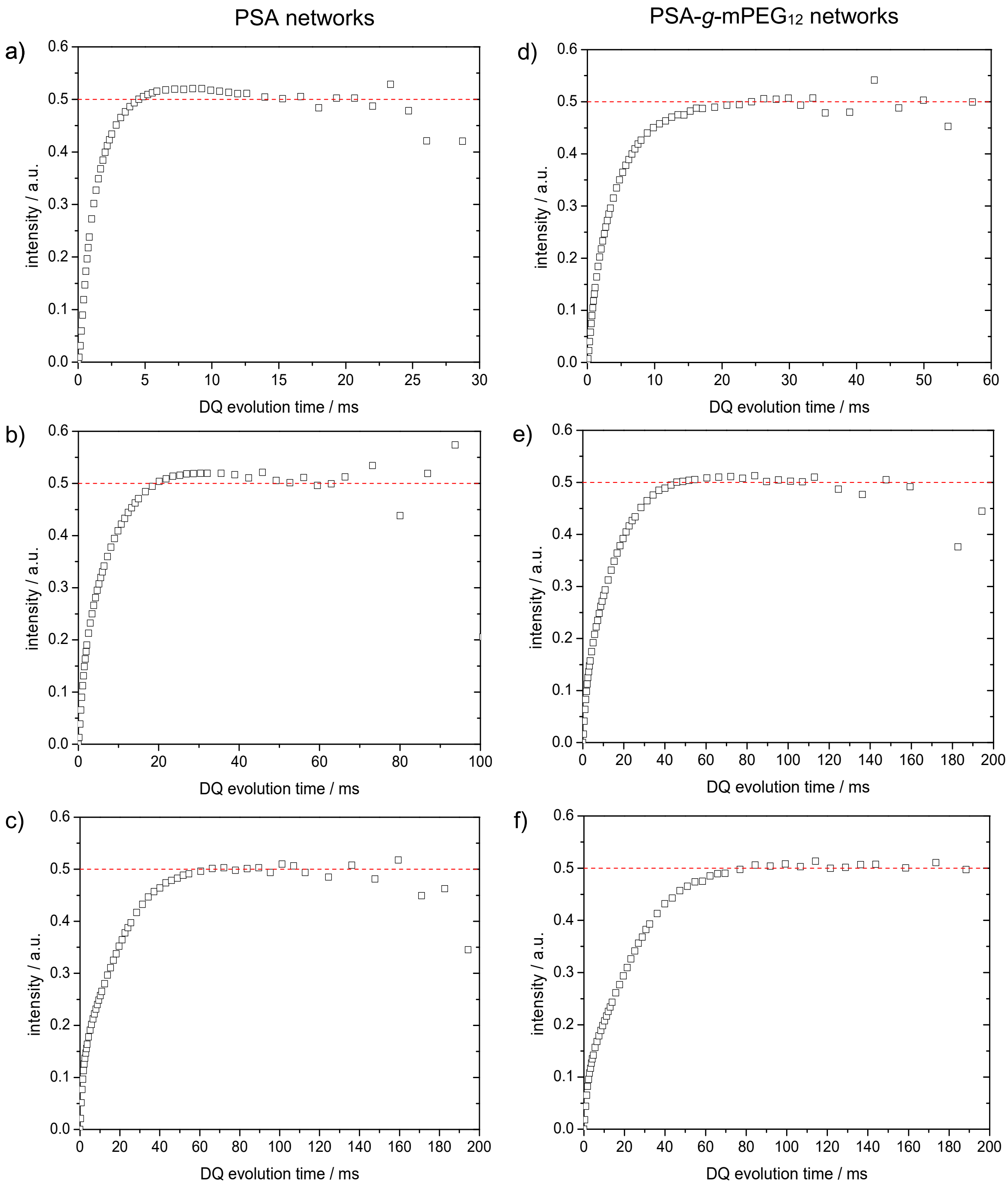

Figure S8. Normalized double quantum curves *nDQ* through ^{1}H DQ NMR for (a-c) PSA networks cross-linked with the Suc-PEG$_n$-Suc (where n = 9, 23, 45) and (d-f) PSA-*g*-mPEG$_{12}$ networks cross-linked with the Suc-PEG$_n$-Suc (where n = 9, 23, 45).

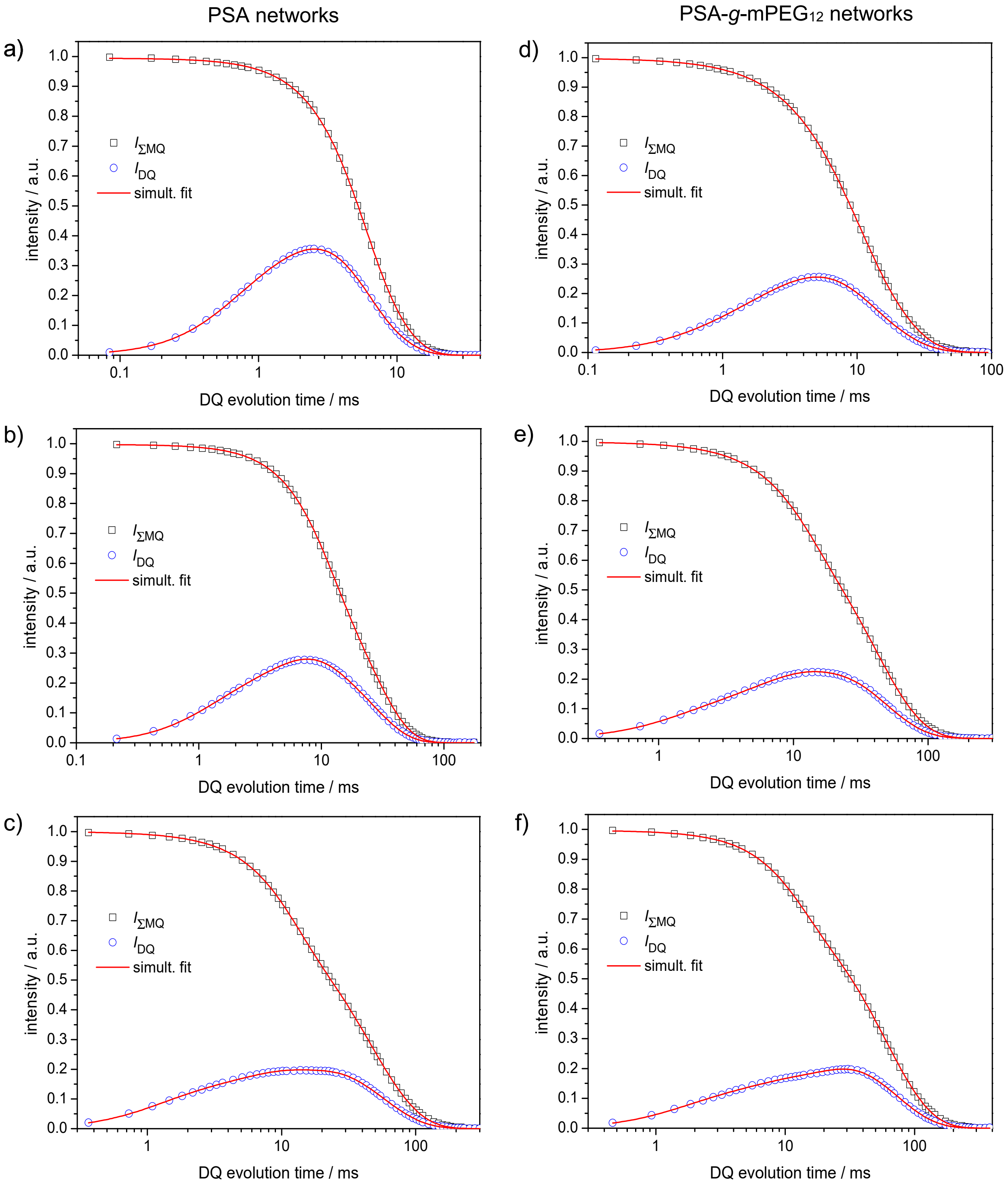

Figure S9. Simultaneous fitting to the sum intensity I_{Σ} and DQ intensity I_{DQ} through ^{1}H DQ NMR after tail correction for (a-c) PSA networks cross-linked with the Suc-PEG$_n$-Suc (where n = 9, 23, 45) and (d-f) PSA-*g*-mPEG$_{12}$ networks cross-linked with the Suc-PEG$_n$-Suc (where n = 9, 23, 45).

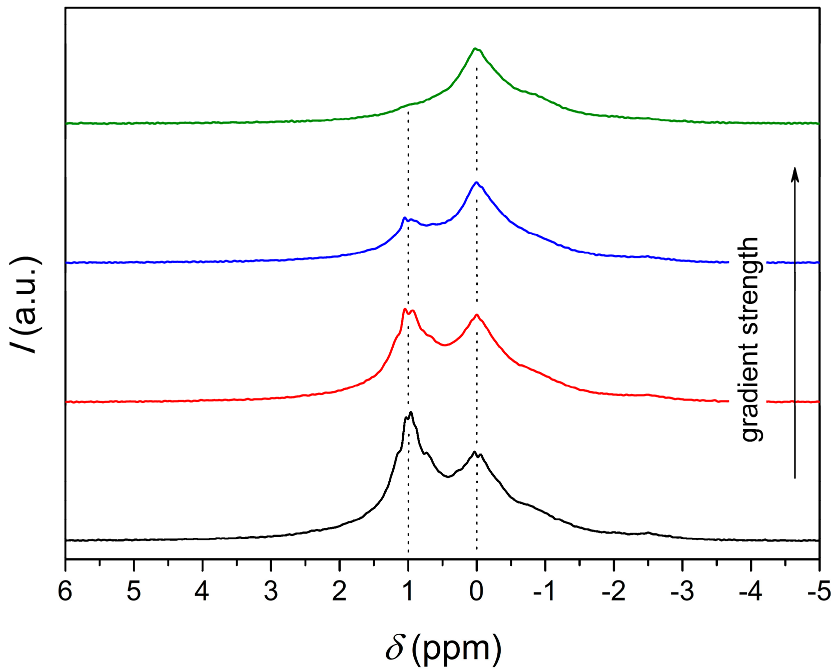

Figure S10. ^{1}H NMR spectra measured with PFG NMR spectroscopy of PSA-*g*-mPEG12 network (Suc-PEG9-Suc) with Q = 6.2 and D_2O as solvent by varying field gradient strength at T = 30°C. The spectrum was referenced to the center of the right peak.

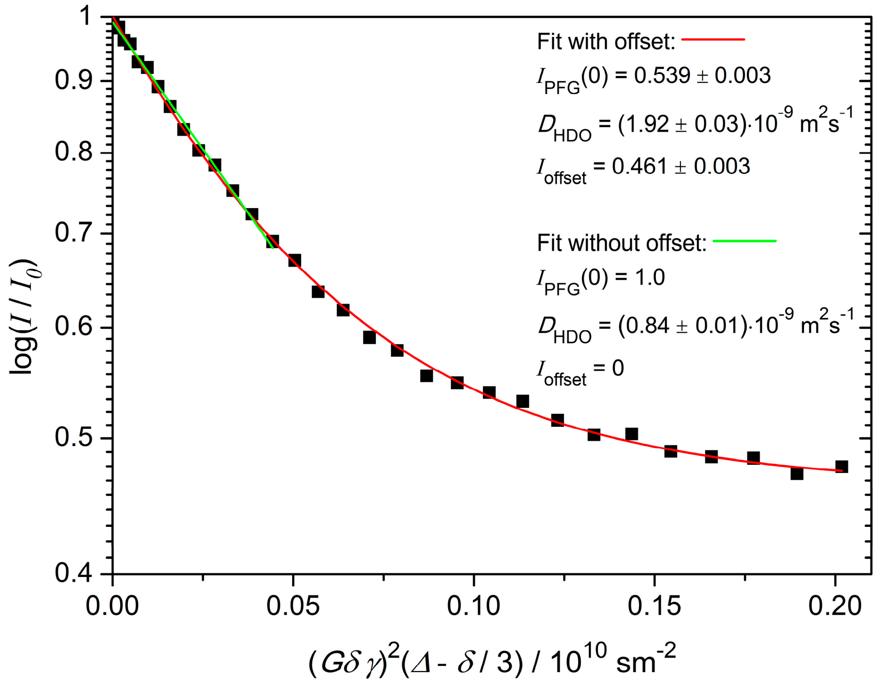

Figure S11. Fit example for the network sample cross-linked with Suc-PEG45-Suc according to eq.2. Different fit strategies for estimation of the diffusion coefficient were tested. The red line: fit with constant offset of 46 % provides D_{HDO} = (1.92 ± 0.03)·10^{-9} $m^2 \cdot s^{-1}$. The green line: fit of the initial decay (linear part of the decay) without offset, which provides D_{HDO} = (0.84 ± 0.01)·10^{-9} $m^2 \cdot s^{-1}$. This value is about 50% lower and corresponds to the arithmetic average of the first value and the offset (D_{offset} = 0).

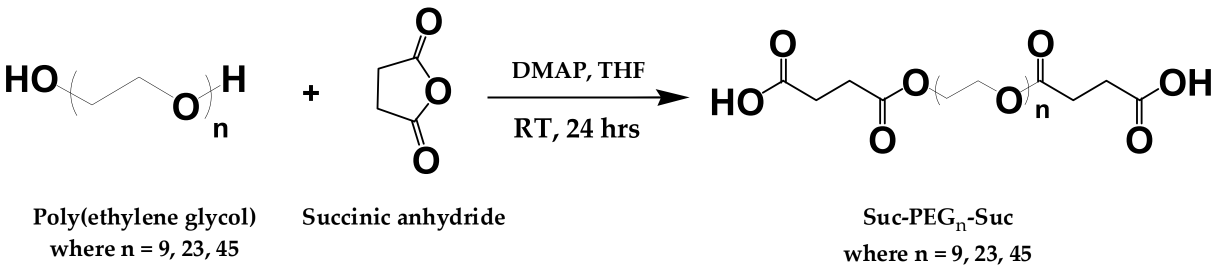

Scheme S2. Synthesis scheme of Suc-PEG$_n$-Suc.

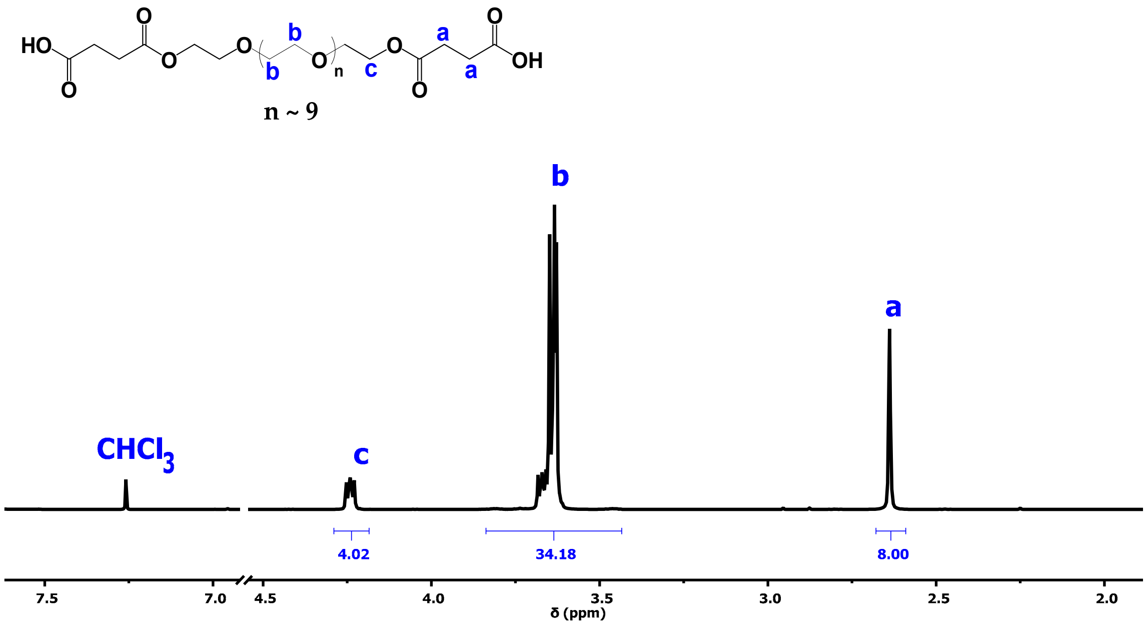

Figure S12. ^{1}H NMR spectrum of Suc-PEG$_9$-Suc measured at 27°C using $CDCl_3$ as solvent.

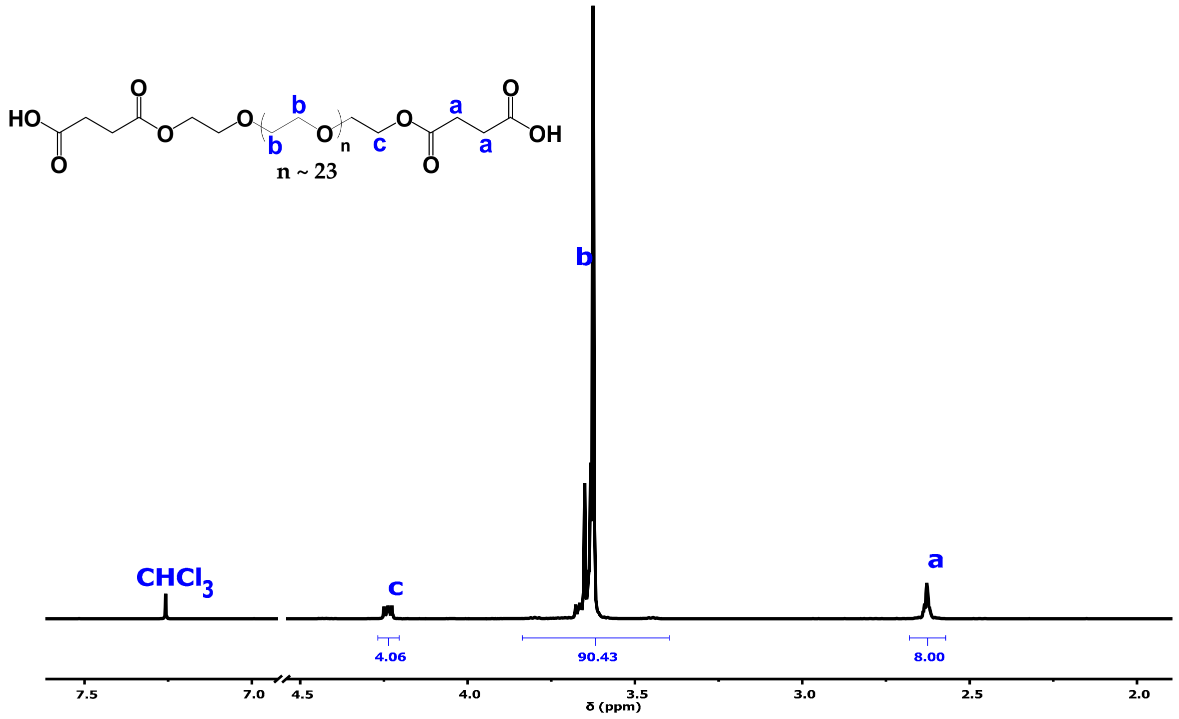

Figure S13. ^{1}H NMR spectrum of Suc-PEG23-Suc measured at 27°C using $CDCl_3$ as solvent.

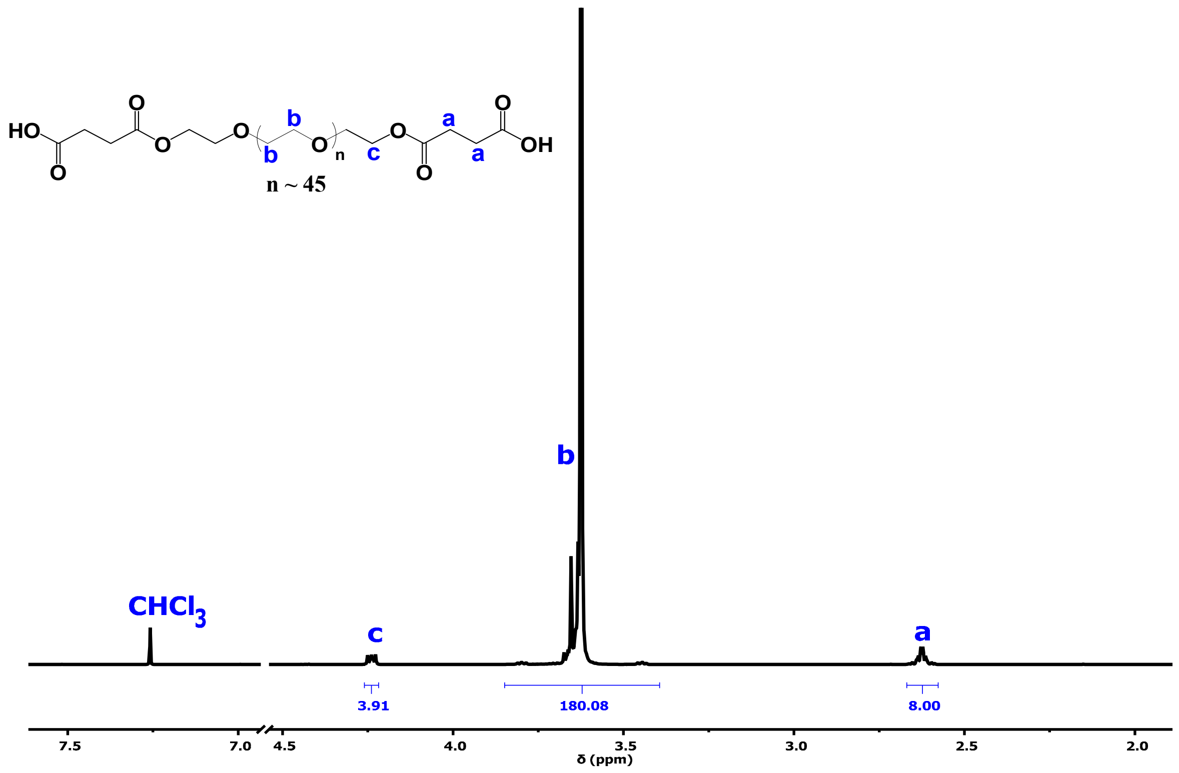

Figure S14. ^{1}H NMR spectrum of Suc-PEG45-Suc measured at 27°C using $CDCl_3$ as solvent.
